# Optimizing the selection of quantitative traits in plant breeding using simulation

**DOI:** 10.3389/fpls.2025.1495662

**Published:** 2025-02-10

**Authors:** Rafael Augusto Vieira, Ana Paula Oliveira Nogueira, Roberto Fritsche-Neto

**Affiliations:** ^1^ Department of Research & Development, Crop Science - Breeding, Uberlândia, Brazil; ^2^ Institute of Biotechnology, Graduate Program in Genetics & Biochemistry and Graduate Program in Agronomy, Federal University of Uberlândia, Uberlândia, Brazil; ^3^ Department of Plant, Environmental and Soil Sciences, Louisiana State University, Baton Rouge, LA, United States

**Keywords:** genetic gain, genetic diversity, simulation, genomic selection, prediction accuracy, selection response, breeding methods, breeding optimization

## Abstract

This review summarizes findings from simulation studies on quantitative traits in plant breeding and translates these insights into practical schemes. As agricultural productivity faces growing challenges, plant breeding is central to addressing these issues. Simulations use mathematical models to replicate biological conditions, bridging theory and practice by validating hypotheses early and optimizing genetic gain and resource use. While strategies can improve trait value, they reduce genetic diversity, making a combination of approaches essential. Studies emphasize the importance of aligning strategy with trait heritability and selection timing and maintaining genetic diversity while considering genotype-environment interactions to avoid biases in early selection. Using markers accelerates breeding cycles when marker placement is precise, foreground and background selection are balanced, and QTL are effectively managed. Genomic selection increases genetic gains by shortening breeding cycles and improving parent selection, especially for low heritability traits and complex genetic architectures. Regular updates of training sets are critical, regardless of genetic architecture. Bayesian methods perform well with fewer genes and in early breeding cycles, while BLUP is more robust for traits with many QTL, and RR-BLUP proves flexible across different conditions. Larger populations lead to greater gains when clear objectives and adequate germplasm are available. Accuracy declines over generations, influenced by genetic architecture and population size. For low heritability traits, multi-trait analysis improves accuracy, especially when correlated with high heritability traits. Updates including top-performing candidates, but conserving variability enhances gains and accuracy. Low-density genotyping and imputation offer cost-effective alternatives to high-density genotyping, achieving comparable results. Targeting populations optimizes genetic relationships, further improving accuracy and breeding outcomes. Evaluating genomic selection reveals a balance between short-term gains and long-term potential and rapid-cycling genomic programs excel. Diverse approaches preserve rare alleles, achieve significant gains, and maintain diversity, highlighting the trade-offs in optimizing breeding success.

## 1 Introduction

Global agriculture faces a convergence of critical challenges, including food and fuel crises, climate fluctuations, and a range of biotic and abiotic stresses. These factors collectively threaten crop yields, creating significant risks to food security and agricultural sustainability. Addressing these challenges requires innovative strategies that consider the complex interactions between genetic, environmental, and economic factors. By deepening our understanding and management of these stresses, we can develop resilient agricultural systems capable of withstanding the pressures of modern crop production.

The growing population and urgent demand for food security necessitate significant increases in both yield potential and crop quality, nutritional composition of agricultural products. This challenge is compounded by shifting consumption patterns and climate variability that further strain agricultural productivity. The need for advancements in crop productivity and resilience is indispensable and can be met, in part, by developing crop varieties and hybrids that thrive in different climatic conditions without sacrificing yield. By harnessing genetic diversity and employing innovative techniques, plant breeding plays an important role in this effort.

The evolution of plant breeding, from early domestication to modern precision breeding, showcases the synergy of scientific inquiry, technological progress, and agricultural practice. By integrating cutting-edge techniques—such as marker-assisted selection, genomic selection, selection indexes, gene editing, artificial intelligence, process automation, data analytics, epigenomics, phenomics, enviromics, and biotechnology—plant breeding has transformed into a precise science. It is now a powerful engine for innovation, driving the development of high-performing crop varieties and addressing evolving agricultural challenges.

## 2 Plant breeding strategies and objectives

Plant breeding is a continuous process focused on improving genetic traits through systematic selection and crossbreeding. A modern plant breeding program typically involves: (ii) selecting individuals with desirable traits for crossbreeding; (i) employing methods to achieve homozygosity, either fully or partially; and (iii) utilizing recurrent selection to recycle high-merit individuals while evaluating them in field trials. This dual strategy ensures the development of new cultivars with improved yield, performance, disease resistance, and quality while continuously improving the genetic foundation of the breeding population.

The simplest selection method is based on phenotypic values, but relying solely on field trials to estimate breeding values is risky, as they may not fully represent the target environment. In this context, since the breeding value determines an individual’s genetic contribution to future generations, effective selection depends on how well phenotype can represent breeding values. To select superior genotypes with high agronomic performance across diverse environments, breeders must test numerous candidates either through multi-year, multi-location phenotypic trials or by genotyping to estimate breeding values. However, many agronomic traits are complex, influenced by multiple quantitative trait loci (QTL), and their expression is heavily impacted by environmental factors.

Genomic estimated breeding values (GEBVs) and genomic selection offer new opportunities to increase breeding efficiency. GEBVs combine genomic and phenotypic data to predict an individual’s genetic merit for specific traits. In genomic selection, candidates are genotyped to obtain GEBVs, bypassing the need for phenotyping. GEBVs are derived from a prediction equation based on the genetic proximity of candidates to a training population, which has been both genotyped and phenotyped.

Genomic predictions rely on accurate estimates of genomic relationships. Implementing genomic selection begins with creating a training population that provides phenotypic data for target traits and genome-wide DNA marker genotypes (Meuwissen et al., 2001). The genotype and phenotype data develop a prediction equation that fits each marker’s effect on the trait. When markers are in sufficient linkage disequilibrium with causal variations of the trait, they capture a significant proportion of the associated genetic variance, allowing the prediction of GEBVs without phenotype collection. Many breeding programs today incorporate genomic data into various phases, improving selection accuracy, shortening breeding cycles, and accelerating early-generation selection (Hickey et al., 2014).

Several strategies integrate genomic selection into breeding programs. For example, rapid-cycle recurrent genomic selection promises significant increases in genetic gains (Santantonio et al., 2020). Combining genomic selection with speed breeding can further reduce breeding cycle time. Genome editing introduces new variations, while marker-assisted selection and transgenic methods enhance breeding efficiency. Nonetheless, effective plant improvement requires balancing genetic gain with time and cost (Hickey et al., 2014). Whether employing phenotypic, genomic, or combined selection approaches, optimizing strategies is crucial to maximizing genetic gain while managing costs and operational efficiency.

Improving multiple traits simultaneously is a key challenge in plant breeding, as the product’s value often depends on interrelated traits. Trait selection methods include tandem selection, which sequentially improves traits across generations, and independent culling, which rejects individuals failing predefined standards for any trait. A widely used approach is multi-trait selection through a selection index. With genomic selection, multi-trait analysis enhances breeding accuracy, especially for low heritability traits linked to high heritability ones (Hayashi and Iwata, 2013).

Assuming clear breeding objectives, rigorous selection criteria, a diverse pool of germplasm, and well-defined target environments, the efficacy of a breeding program predominantly depends on the efficient utilization of available resources to respond to selection, also known as genetic gain. Optimizing either genomic or phenotypic selection approaches within breeding pipelines is crucial. Substantial genetic progress requires considering several factors, including the number of breeding cycles, population size, parent selection, genomic prediction accuracy, genetic diversity, and cost management.

## 3 Simulations and their role in plant breeding

### 3.1 Definition and applications

**Figure 1:**
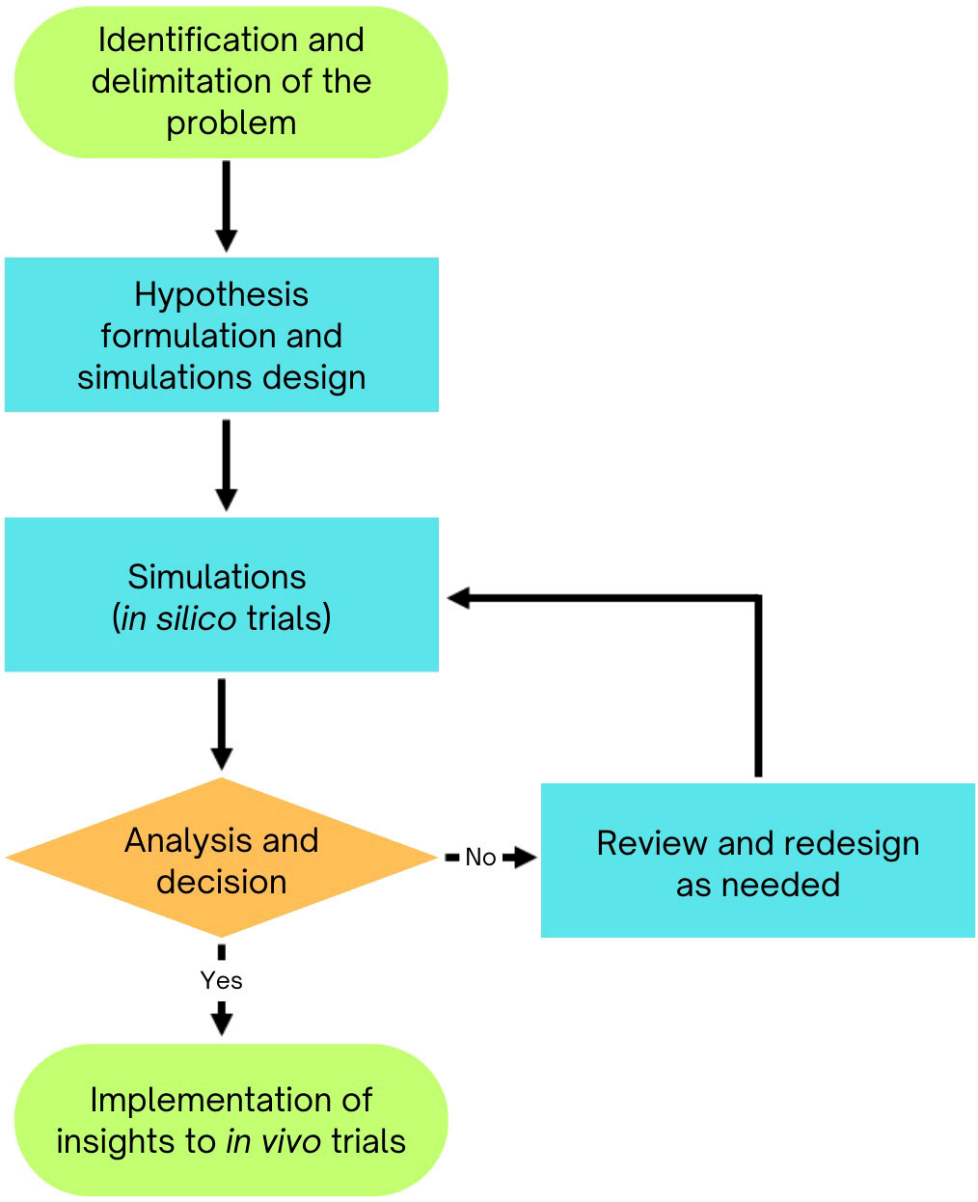
Diagram of simulation models in plant breeding, illustrating how mathematical models replicate biological conditions to bridge theoretical concepts with practical breeding applications.

In plant breeding and genetics, simulations use mathematical models to replicate biological conditions and investigate specific problems (**Figure 1**). These models are either deterministic or stochastic (Covarrubias-Pazaran et al., 2022). Deterministic models rely on equations from quantitative genetics to predict selection responses using parameters like selection intensity, heritability, and accuracy. However, they have limitations in accounting for breeding processes such as crossing, generation advancement, and genetic introgression. Stochastic simulations, on the other hand, generate genotypic and phenotypic data for each genetic entity, making them more applicable to breeding stages like recombination, evaluation, and selection.

Simulations bridge theory and practice by computationally modeling breeding strategies to optimize genetic gain, minimize genetic variance loss, and ensure resource efficiency. They compare strategies like phenotypic, marker-assisted, and genomic selection over various timeframes, incorporating early- and late-stage processes. Simulations also identify optimal selection factors, such as intensity, parental lines, and family sizes, while considering genotype-by-environment interactions, inheritance, and gene effects. By validating hypotheses prior to real-world testing, simulations streamline transitions from phenotypic to marker-assisted and genomic selection.

### 3.2 Limitations and advantages

One limitation of breeding scheme simulations is the potential for random error, reflecting real-life processes like meiosis and genotype evaluation, where simulations often introduce random deviations from the genotypic value (Jannink et al., 2024). These simulations can also be computationally intensive. Genomic predictions require large-scale linear mixed models, which are time-consuming for both real and simulated data. Incorporating training data from previous selection cycles is possible, but computing limitations may arise when dealing with large datasets (Ramasubramanian and Beavis, 2021).

Simulation accuracies often exceed real-world conditions due to factors like error-free molecular markers, absence of epistasis, and limited germplasm exchange (Gaynor et al., 2017). Therefore, careful interpretation is necessary when applying simulation results to practical breeding. Real-world breeding programs also account for multiple traits—such as agronomic performance, disease resistance, and end-use quality—adding complexity to simulations, which must represent multi-trait selection accurately.

Despite these challenges, simulations offer significant advantages. They allow the exploration of various scenarios, genetic models, and methods, helping identify efficient paths to target cultivars (Li et al., 2012). This is particularly important in genomic selection, where balancing resources, program size, and genetic gain is critical. Simulations provide insights into factors affecting genetic gain, prediction accuracy, and cost-effectiveness under different conditions (Hickey et al., 2014; Gorjanc et al., 2018).

While field trials are essential, they provide only a snapshot of outcomes, and random effects may distort results (Li et al., 2012). Simulations, with multiple replications, offer more reliable probabilistic assessments and can test a range of input parameters to evaluate their impact on selection efficiency. They also account for multistage selection and inbreeding rates (Hassanpour et al., 2023).

Modern simulation is characterized by the capacity to model meiotic processes, including crossing over and crossover interference, through coalescent and gene-drop methods, which are employed in backward-in-time and forward-in-time simulations (Hickey and Gorjanc, 2012). The coalescent method generates whole-chromosome founder haplotypes with linkage disequilibrium and allele frequency that align with a specified population genetic model (Hickey and Gorjanc, 2012). Conversely, the gene-drop method simulates the creation of new haplotypes from original founder haplotypes by modeling genetic recombination during meiosis. This process is guided by a genetic map and incorporates the gamma model, which accounts for crossover interference (Hickey and Gorjanc, 2012).

Overall, simulations have become crucial in plant breeding, enabling the evaluation of genetic gain and comparison of breeding strategies by incorporating gene information, crossing schemes, population size, and selection intensity. This helps optimize breeding parameters to maximize gains, while efficiently allocating resources (Li et al., 2012; Hickey et al., 2014; Gorjanc et al., 2018). Simulations also aid in gene mapping, validating new methods, and assessing factors like marker density and QTL heritability (Li et al., 2012).Translating breeding strategies into digital schemes requires abstraction to represent biological and operational processes while addressing programming limitations. Breeding involves iterative cycles of selection, crossing, and evaluation, which must be encoded into simulations. Challenges include balancing computational efficiency with biological realism, handling large datasets, and modeling diverse genetic architectures. Simulations often assume idealized conditions—error-free markers, no genotype-environment interactions, or perfect linkage maps—which may not align with real-world complexities. Interpreting results requires critical evaluation of these assumptions. Practical integration involves validating insights with field data, incorporating feedback loops, and refining models to align predictions with real-world outcomes. Simulated genetic gain represents a theoretical maximum, often reduced by attrition during practical implementation.

## 4 Diversity, background recovery, pre-breeding and bridging to elite germplasm

A simulated study by Fu (2015) assessed how selection impacts genetic diversity, confirming that plant breeding can reduce diversity as expected. Although artificial selection enhanced the genetic value of targeted traits, it also decreased genetic diversity and heterozygosity. The breeding schemes studied showed similar effects, either preserving or accelerating diversity loss, with half-sib mating proving the most effective for achieving higher genetic gains while minimizing reductions in diversity. The trends in trait improvement and diversity loss were consistent for progeny sizes of 20 and 50, offering important insights for managing these factors.

Gorjanc et al. (2016) investigated different strategies and genetic parameters for initiating pre-breeding programs with selected landrace populations for integration into elite maize breeding programs. Their results indicated that starting pre-breeding with landraces could significantly influence genetic merit. Higher genetic merit was found when the founding population had low diversity, high within-landrace diversity, or substantial heritability (h² = 0.50). Although testcross initiation provided the highest genetic merit, it mainly reconstructed the elite genome and did not incorporate the desired traits from landraces. The authors recommended random mating between initial landrace × elite individuals across several generations to recombine genetic segments and break their linkage. Krenzer et al. (2024) emphasized the importance of simulation-designed pre-breeding crossing schemes for maintaining genetic variation and ensuring long-term success before implementing general combining ability-based selection in hybrid breeding programs.

Building on this, Allier et al. (2020) advanced the understanding of pre-breeding strategies by investigating the integration of new germplasm into elite lines using genomic selection and optimal cross-selection in simulations. Their study introduced donors with various performance levels, showing that recurrent introductions of improved donors could maintain genetic diversity and enhance both mid- and long-term performance. Considering a bridging step resulted significantly higher mid- and long-term genetic gain even when introducing low performing donors. Further, direct introductions of donors with a large performance gap compared to elite germplasm did not generate gains. For donors with an intermediate performance gap, both direct introductions and bridging steps provided increased long-term gains. Additionally, a genomic selection model trained on both bridging and breeding progeny improved prediction accuracy within introduction families. This suggests that excessive selection for performance during the bridging phase might favor the elite genome, limiting the incorporation of new favorable alleles, as noted by Gorjanc et al. (2016).

Cowling et al. (2017), Villiers et al. (2024), and Platten and Fritsche-Neto (2023) highlight the complex balance required between genetic diversity, selection intensity, and long-term genetic gains. Cowling et al. demonstrated the advantages of optimal contribution selection (OCS) over truncation selection, with OCS yielding higher index values and maintaining lower coancestry across various population sizes and selection pressures. Villiers et al., building on the importance of genetic diversity underscored by Cowling et al., introduced optimal haplotype stacking (OHS) as a superior strategy for preserving diversity while achieving genetic gains. Their results, particularly in small populations, revealed that even a single OHS cycle could outperform OCS and truncation selection in long-term outcomes, emphasizing its potential as a complement to other methods.

Platten and Fritsche-Neto (2023) further nuanced this discussion by focusing on strategies to integrate new genetic material into breeding programs. Their findings showed that while introducing fewer parents (e.g., 10 rather than 20) minimized penalties and enhanced fixation efficiency, genetic diversity and background recovery remained critical factors. This aligns with Villiers et al.’s emphasis on diversity preservation and Cowling et al.’s observation of plateauing gains under traditional truncation selection. Notably, Platten and Fritsche-Neto’s recommendation to prioritize GEBV-based selection parallels the targeted approaches of OCS and OHS, highlighting how tailored strategies optimize genetic outcomes under different constraints. Together, these studies illustrate the trade-offs inherent in balancing genetic diversity, selection efficiency, and long-term genetic progress. While Cowling et al. advocate for OCS in avoiding diversity erosion over extended cycles, Villiers et al. demonstrate how OHS can bolster diversity and gains in the short term, even when combined with truncation selection. Meanwhile, Platten and Fritsche-Neto emphasize the necessity of strategic parent selection to achieve rapid fixation of desirable traits. In conclusion, balancing genetic gain and diversity remains a critical challenge in plant breeding. Strategies like optimal contribution selection and optimal haplotype stacking show potential to address this issue. Integrating landraces or other germplasm donors with good performance can improve long-term gains, but careful planning is necessary. Ultimately, combining strategic selection approaches is crucial for optimizing breeding outcomes while maintaining genetic variability, as simplistic methods are inadequate for navigating this complex process.

**Figure 2:**
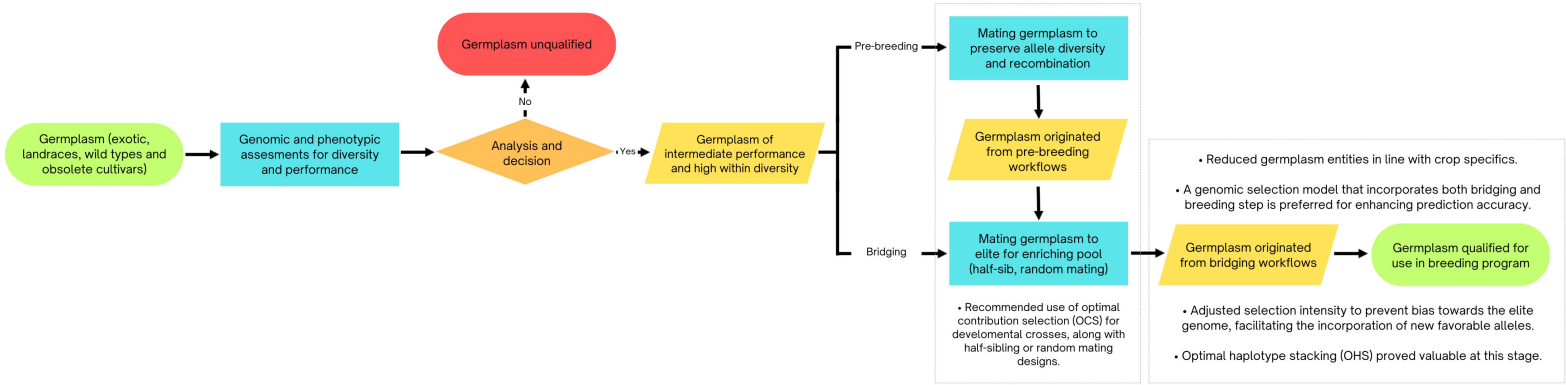
Flowchart depicting the application of simulation-derived strategies for managing genetic diversity, pre-breeding processes, and transitioning from theoretical models to elite germplasm.

The findings from this section are illustrated in **Figure 2**.

## 5 Phenotypic selection and classical methods, including gene effects

Casali and Tigchelaar (1975) conducted pioneering simulations to investigate breeding methods, focusing on single seed descent (SSD) combined with pedigree and mass selection. Their research found that SSD, whether used alone or with mass selection, better preserved genetic variability for line selection in the F6 generation. Pedigree and mass selection were effective for high heritability traits in early generations (F2 and F3), while SSD and F6 selection maximized genetic gain for low heritability traits. Their work demonstrated that early generations are effective for traits with medium or high heritability, while traits with low heritability benefit from later selection. These findings laid the groundwork for modern breeding practices.

Complementary, Van Oeveren and Stam (1992) examined breeding strategies for self-pollinating crops using simulations. They compared early selection, which estimates cross means and variances in the F3 generation and selects lines based on these estimates, with SSD, which delays selection until the F6 generation. SSD performed comparably to early selection with more crosses and outperformed early selection at low heritability due to early prediction inaccuracies. Differences between early selection and SSD decreased when traits were influenced by many loci. SSD was less effective with fewer than 100 F2 plants, and the number of segregating loci had a significant impact on selection outcomes.

These studies underline a critical principle in plant breeding: the choice of breeding strategy should be matched to trait heritability and selection timing. Early selection methods are advantageous for traits with high heritability, while SSD is more effective for traits with low heritability when selection is delayed. They highlight the importance of maintaining genetic diversity and considering genotype-environment interactions to avoid biases in early phenotypic selection. These findings were based on simulated data and predate the adoption of marker-assisted and genomic selection techniques.

Building on this, Wang et al. (2003) compared two prevalent breeding strategies—modified pedigree selection and selected bulk selection—using genetic models that accounted for epistasis, pleiotropy, and genotype-environment interactions. Their simulations indicated that bulk selection achieved a 3.3% higher genetic gain than modified pedigree selection, required one-third less land, and produced 60% fewer families, highlighting its superior efficiency. The study emphasized that selected bulk selection not only resulted in marginally higher genetic gains but also offered significant cost-effectiveness advantages, which is crucial for modern commercial breeding programs.

In a subsequent study, Wang et al. (2004) examined the impact of partial dominance in wheat and found that it had minimal effect on genetic advancement compared to pure additive models. The partial dominance effects were small and difficult to detect through covariance across various mating schemes, suggesting limited applicability in inbred breeding. These observations may be specific to wheat or reflect limitations in the simulations’ ability to capture such gene effects. Additionally, Wang et al. (2007) compared three marker selection schemes for integrating nine genes into a single genotype and recommended a top cross scheme. This approach, involving equal selection intensity at three stages (top cross F1, top cross F2, and doubled haploid), minimized the total number of lines needed to achieve the breeding objective.

Continuing the focus on maximizing selection response and genetic gain, Bernardo (2003) analyzed the impact of parental selection, the number of breeding populations, and population size on optimal breeding strategies. Using computer simulations with 2,000 recombinant inbreds for a quantitative trait controlled by 100 additive loci with varying heritabilities, Bernardo found that the highest selection responses were achieved by maximizing the number of breeding populations. The ability to identify high-performing breeding populations before making crosses was more critical than balancing the number of populations and their size.

As emphasized by Bernardo, successful breeding depends on strategic parental selection before establishing breeding populations. A widely accepted recommendation is to use genetically divergent yet superior parents to broaden the genetic base and enhance target traits.

Covarrubias-Pazaran et al. (2022) simulated various parent combinations to provide practical recommendations for breeding programs. Their model, designed for cassava but applicable to other crops, found that overlapping cohorts for recycling—using a mixed crossing block with parents from both preliminary and advanced yield trials—consistently led to higher genetic gains. However, the number of parents used was critical; using more than 30 parents reduced genetic gain compared to fewer. For 60 years, involving 16 to 32 parents proved most effective, with more crosses generally enhancing genetic gains. Within a 20-year timeframe, fewer crosses with more progeny per cross yielded higher gains, with an optimal setup being 8–16 parents, 24 crosses, and approximately 68 progenies per cross annually. Over 60 years, the optimal configuration was 16–32 parents, 60 crosses, and around 30 progeny per cross. The study recommended using 15–30 parents recycled from both preliminary and advanced yield trials, with 40 crosses and 40 progenies per year, a strategy that could be adapted to other crops.

Supporting these findings, Lanzl et al. (2023) demonstrated through simulations that mating designs producing large biparental families from few disjoint crosses risk generating progenies with strong covariances between QTL pairs on different chromosomes. Their research revealed that a single recombination round effectively disrupts both positive and negative covariances between QTL pairs, providing practical insight for optimizing breeding programs.

In summary, optimizing genetic gain in breeding programs involves strategic parental selection and careful management of breeding populations. Using overlapping cohorts for recycling, along with an optimal number of parents and crosses, can significantly enhance genetic gain. Identifying high-performing breeding populations prior to initiating crosses is essential. Additionally, the optimal number of parental lines involved in breeding crosses varies depending on the breeding timeframe. Specifically, a larger number of parents is beneficial for achieving long-term genetic gains, whereas fewer parents are recommendable for attaining higher short-term, immediate gains. These strategies, validated through simulations, apply to both phenotypic and genomic approaches, improving breeding efficiency across various crops.

Backcross is a common method in plant breeding for transferring specific traits from one genetic entity to another while retaining desirable characteristics of the original line. Another common approach in developing breeding crosses involves emphasizing the importance of one parent over the other. In this method, the F1 generation is backcrossed with the superior parent to enhance its desirable traits, either to recover the parental genetic background or directly derive recombinant lines for the development of new cultivars.

Wang et al. (2009) used computer simulations to show that single backcrossing with a selected bulk is more effective than other crossing and selection methods. This approach is particularly useful for preserving or enhancing the adaptation of recurrent parents while transferring most of the desired donor genes. However, repeated backcrossing may not be necessary if molecular markers can identify the transferred genes, especially when donor parents exhibit poor adaptation. The study identified three conditions under which single backcrossing with bulk selection until F6 is highly effective: (1) when traits being transferred are controlled by multiple genes, (2) when donor parents have genes that can improve adaptation in recipient parents, and (3) when conventional phenotypic selection is used, or individual genotypes cannot be easily identified. These insights are valuable for introgressing elite germplasm in rapid-cycle breeding programs, enhancing both efficiency and outcomes.

Continuing on this topic, backcrossing is used to introduce traits or genes into important crops, such as corn. This process includes four stages: single-event introgression, event pyramiding, trait fixation, and version testing. The goal is to ensure that at least one version of the final product performs as well as the original. Sun and Mumm (2015) investigated two critical aspects of this process through simulations: (i) the impact of residual donor germplasm (non-recurrent parent) in the converted germplasm and (ii) the effect of creating multiple versions for each parental line conversion. Their research found that a residual donor germplasm range of about 5-8% of the genome was associated with a high success rate in conversion. Additionally, the success rate increased with the number of versions produced, with 3-5 versions providing the best outcomes. These findings offer practical insights for market-assisted backcrossing in other crops, emphasizing the importance of managing residual donor germplasm and generating multiple versions during germplasm conversion for specific traits.

**Figure 3:**
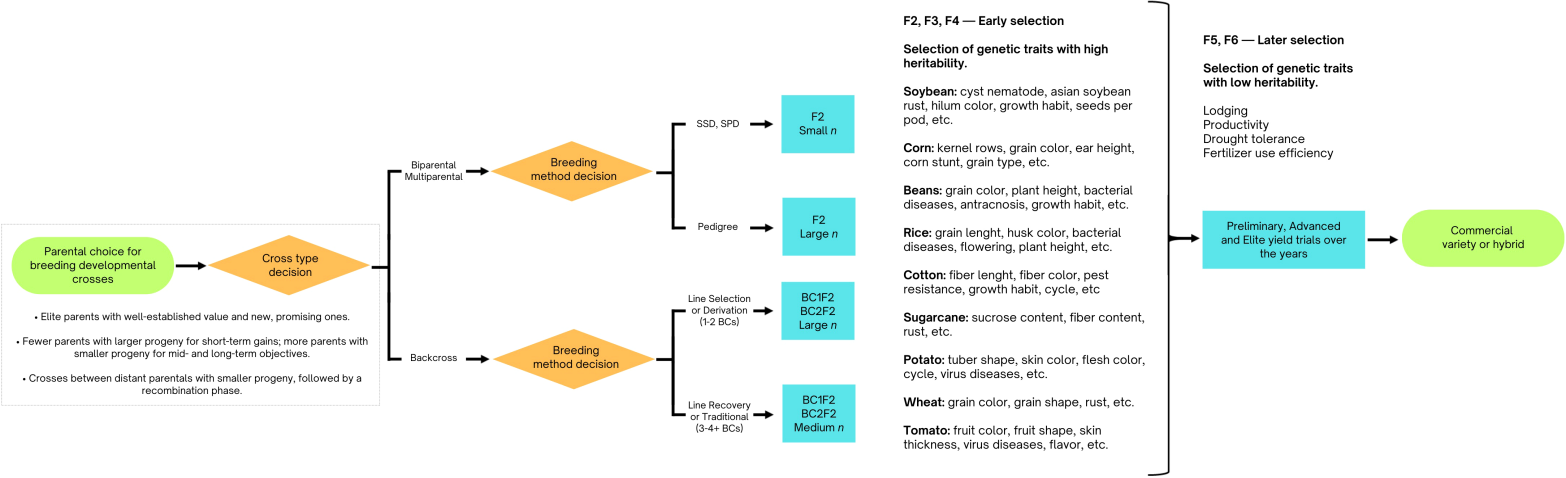
Flowchart demonstrating practical insights for phenotypic selection and traditional breeding methods, as informed by simulation studies. It outlines how these methods can be optimized to balance trait selection and genetic diversity.

The findings from this section are illustrated in **Figure 3**.

## 6 Marker-assisted selection methods and QTL studies

In the early development of marker-assisted selection (MAS), simulations were primary utilized to assess its feasibility as either a complement or replacement for conventional breeding methods. The secondary goal was to optimize MAS integration into breeding strategies for both allogamous and autogamous crops.

Simulation studies by Edwards and Page (1994) and Van Berloo and Stam (2001) assessed the effectiveness of MAS compared to phenotypic selection. They investigated various genetic structures, parental populations, and crop species with different reproductive systems. At this point in history, when researchers assessed the potential of applying MAS to quantitative traits, studies found that while MAS led to rapid initial gains, these improvements diminished significantly after three to five cycles. MAS was particularly effective in utilizing genetic diversity in more heterozygous populations, outperforming phenotypic selection. However, its effectiveness was limited by linkage disequilibrium between markers and QTL, being effective for traits controlled by fewer QTL, such as disease resistance and growth habits in autogamous plants, but not yield.

Despite these limitations, MAS at that time offered notable advantages, including the ability to perform two selection cycles per year for faster initial improvements, with conventional methods potentially taking over in later stages (Edwards and Page, 1994). MAS also provided more accurate predictions of superior parental combinations for multiple traits compared to phenotypic selection (Van Berloo and Stam, 2001). Thus, while MAS generally outperformed phenotypic selection, its initial gains highlighted the value of integrating MAS with traditional breeding methods to maximize genetic potential.

Hospital et al. (1997) further explored MAS through simulations and found that its response at low heritability levels was more variable compared to phenotypic selection. Nonetheless, MAS’s efficiency increased with larger population sizes, even for low heritability traits. MAS was effective in fixing QTL with large effects in early generations. However, while fixing QTL alleles with small effects, the advantage was offset by a higher fixation rate of unfavorable background alleles or an increased likelihood of linkage drag, making MAS less efficient than phenotypic selection over time. MAS operates by targeting favorable alleles linked to specific markers, independent of a trait’s heritability (h²). Nevertheless, heritability indirectly affects the efficacy of MAS, as it reflects the proportion of phenotypic variation explained by genetic factors. Traits with high h² facilitate more precise marker-trait associations, enhancing the reliability of selection, whereas traits with low h² may experience reduced accuracy due to a stronger influence of environment. Consequently, while MAS is not directly contingent on h², the strength of the genetic signal remains a determinant of its success.

Overall, these studies emphasize MAS’s substantial initial benefits in plant breeding, particularly in capturing genetic diversity and facilitating rapid gains in early selection cycles. MAS is especially effective in more heterozygous populations and for specific traits such as disease resistance. However, the reliance on linkage disequilibrium between markers and QTL presents challenges, limiting MAS’s effectiveness for complex traits like yield.

In managing and optimizing marker-assisted selection (MAS), Lande and Thompson (1990) highlighted that the effectiveness of MAS and the advantages of molecular markers depend on genetic parameters and the selection strategy used. They emphasized that large population sizes can enhance the detection of additive genetic variance through marker associations, accelerating quantitative trait improvement.

Defining practical population sizes is essential for cost-effective breeding. Ooijen (1992) investigated the use of adjacent marker pairs to improve QTL linkage information within specific chromosomal segments. The study demonstrated that with 200 backcross or F2 individuals, there is a high probability of identifying QTL accounting for at least 5% of the total variance. Although QTL with larger genetic effects are mapped more precisely, challenges remain for traits controlled by multiple minor effect genes. Ooijen concluded that QTL mapping accuracy is influenced by trait heritability, gene number, interactions, and marker distribution. To enhance QTL effect estimation, he recommended increasing sample sizes and testing environments.

Bernardo and Charcosset (2006) argued that targeting QTL with large effects is more beneficial than including those with smaller effects. Their findings indicated that marker-assisted recurrent selection programs are most effective when focusing on traits controlled by a moderately large number of QTL, approximately 40. For traits governed by fewer genes, having more genetic information correlates with a higher selection response. In contrast, for traits influenced by 40 or 100 genes, including all genes in the selection model results in a lower selection response (Bernardo and Charcosset, 2006). Although these conclusions are now considered intuitive, they were significant in shaping subsequent research and practical MAS applications in plant breeding.

Gimelfarb and Lande (1994) confirmed that using genetic markers can effectively utilize linkage disequilibrium between markers and QTL generated by crossing inbred lines. They cautioned that merely increasing the number of markers does not necessarily improve selection efficiency. Instead, the population size under selection was identified as a more critical factor for MAS efficiency, aligning with Lande and Thompson’s findings (1990). Darvasi and Soller (1997) further refined molecular marker spacing in backcross and F2 linkage experiments through simulations, providing formulas for calculating the optimal number of markers based on factors like population size, and allele substitution effects, and explained variance. Their recommendations offered practical guidance for MAS strategy design.

In autogamous crops, MAS has been shown to improve selection outcomes for obtaining superior genotypes compared to traditional methods (Van Berloo and Stam, 1998). This aligns with earlier findings suggesting MAS can outperform phenotypic selection techniques. Van Berloo and Stam highlighted the advantages of MAS when dominant alleles at QTL are linked in a coupling phase—where loci on the same chromosome are co-inherited more frequently. However, the benefits were moderated by uncertainties in QTL map positions, underscoring the need for precise QTL mapping. Charmet et al. (1999) further supported this by finding that precise QTL location significantly impacts selection when using inbred line parents to maximize favorable allele accumulation. Fine-tuning QTL following initial identification remains crucial in modern crop breeding.

Marker-assisted selection (MAS) offers improved accuracy in parental selection and potentially quicker breeding cycles but tends to be less efficient over time, particularly as the risk of fixing unfavorable alleles increases in later generations. These observations suggest that MAS should complement traditional breeding methods to maximize genetic potential and ensure long-term improvement. Kuchel et al. (2005) investigated integrating restricted backcrossing and doubled haploid (DH) techniques in MAS breeding strategies. They found that applying MAS at all three stages of breeding was effective for achieving high frequencies of desired outcomes and combining favorable traits. However, they identified that applying MAS at two specific stages (BC1F1 and haploid) was optimal. This approach increased genetic gain compared to phenotypic selection and reduced overall costs by 40%.

A prevalent application of MAS is optimizing backcross phases in breeding programs. Early simulations were crucial in refining this method. Viicher et al. (1996) demonstrated that genetic markers were effective for introgressing specific alleles and selecting the desired background in backcross populations derived from inbred lines. Their simulations showed that markers spaced 10–20 cM apart could shorten the breeding process by one to two generations compared to random or phenotypic selection methods. This time reduction is essential for achieving genetic gain and delivering products efficiently.

Hospital and Charcosset (1997) found that for generations 1 to 3, background selection on both carrier and non-carrier chromosomes with equal weights is optimal. Selection for recipient parent marker alleles should include both carrier and non-carrier chromosomes. They indicated that manipulating no more than four QTL simultaneously is advisable, requiring approximately 1600 individuals to achieve a target genotype with 99% confidence, though using a pyramidal design can reduce this number to 580.

Ribaut et al. (2002) similarly demonstrated that the selection response in BC1 is significantly enhanced when the selectable population size is fewer than 50, with diminishing returns above 100. Efficient selection is achieved by targeting loci in BC1 and BC2 combined with background selection in BC3. Specifically, a population size of 10 in BC1 and BC2, and 100 in BC3, can reduce the donor genome to below 5%, which is often desirable. Peng et al. (2014) recommended a selection scheme involving five generations of MAS for integrating up to 15 transgenic events. This involves selecting for the event of interest in BC1 through BC3 with a population size of 600, and for both the event and recurrent parent germplasm in BC4 and BC5 with a population size of 400, using a selection intensity of 1% for all generations.

**Figure 4:**
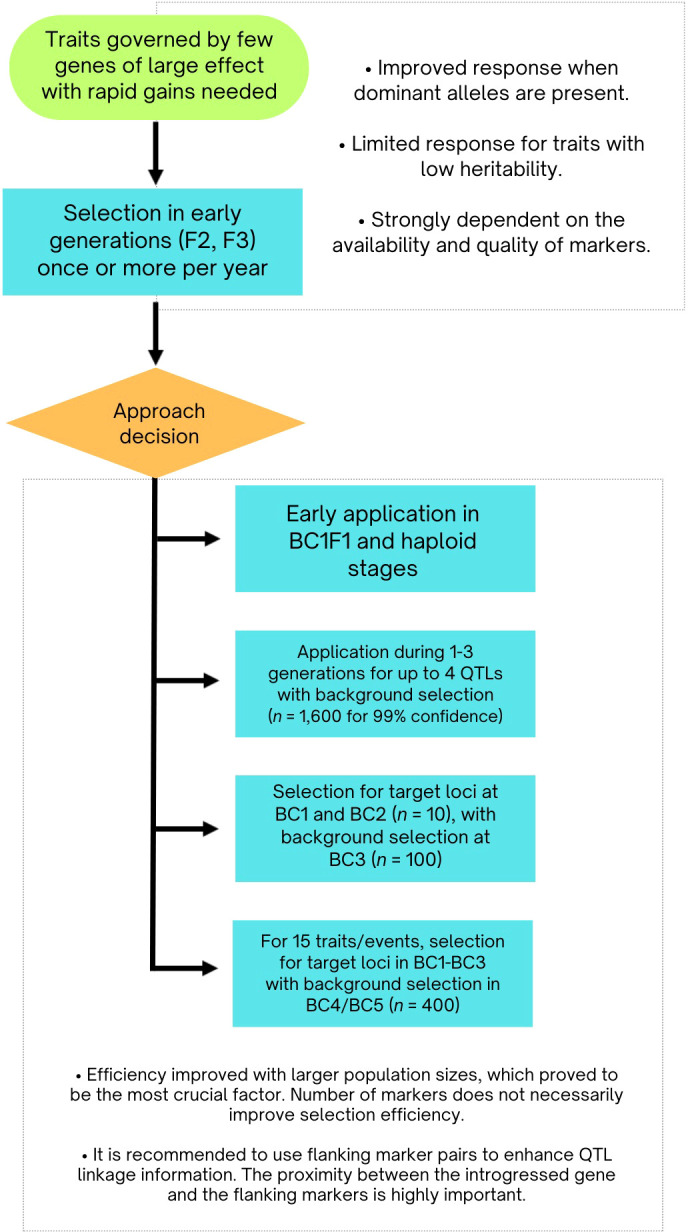
Flowchart showcasing marker-assisted selection and QTL management strategies derived from simulation research.

These findings are summarized in **Figure 4**. The integration of MAS has significantly enhanced the efficiency of backcross breeding programs, particularly for allele introgression and background optimization. Strategic marker use can expedite breeding cycles, improving genetic gains and product delivery. Key aspects include precise marker positioning, balancing foreground and background selection, and managing the number of QTL. Effective background selection in early generations and controlling QTL numbers are essential for maximizing efficiency. Additionally, optimizing selectable population sizes underscores the strategic role of MAS in achieving targeted genetic outcomes.

As noted, the scarcity of marker-assisted selection (MAS) methods and QTL studies in the literature over the past decade reflects the substantial evolution in breeding technologies. This shift is largely attributed to the emergence of genomic selection (GS).

## 7 Genomic selection

**Figure 5:**
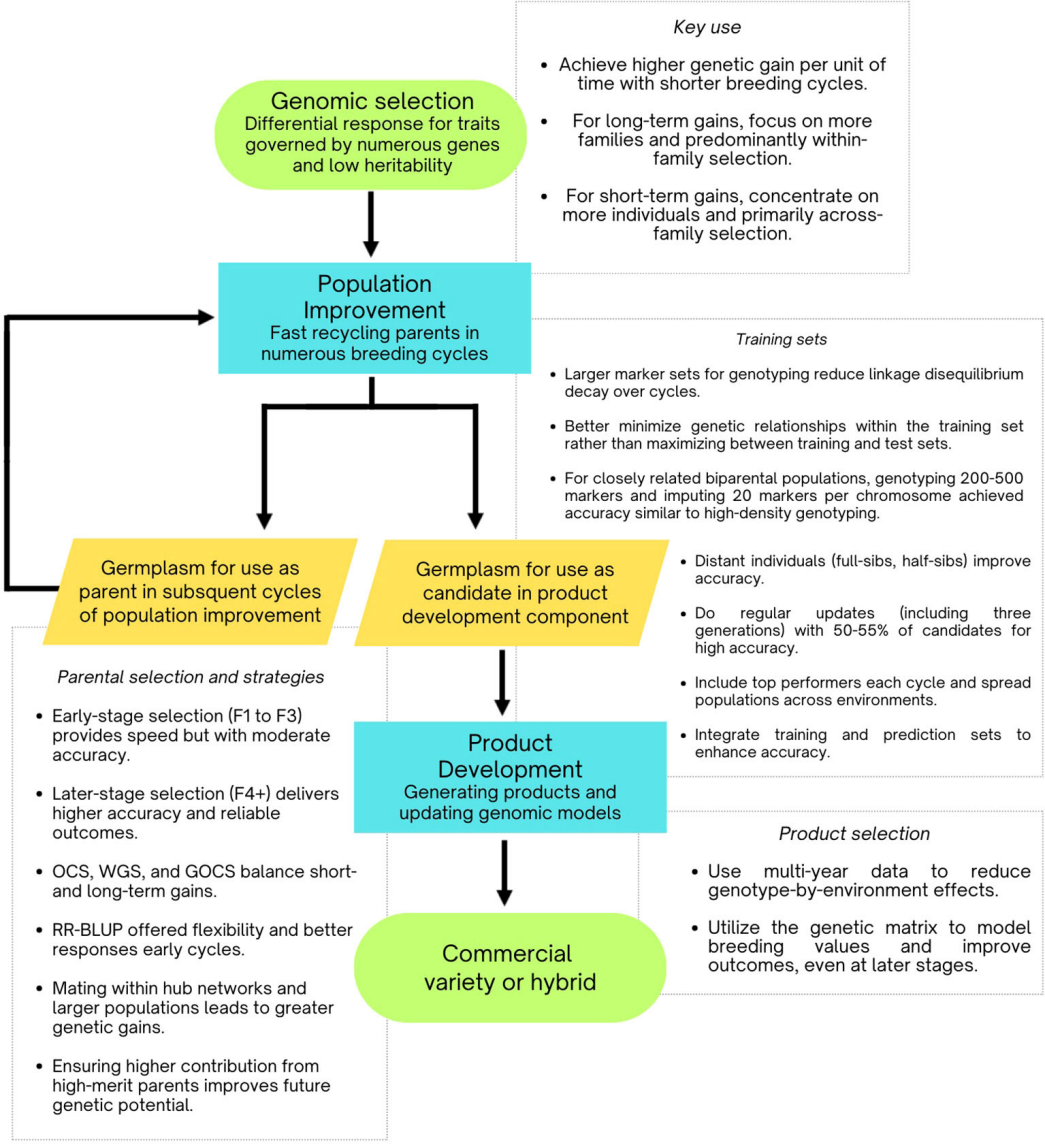
Flowchart illustrating practical schemes for implementing genomic selection, based on insights from simulation studies.

Emerging from the limitations of marker-assisted selection (MAS), genomic selection has become the foundational methodology in modern plant breeding. Since its introduction, computer simulations have been essential in demonstrating and optimizing genomic selection to achieve greater and more sustainable genetic gains. These insights are summarized in **Figure 5**.

Two key simulation-based studies established the framework for genomic selection before the term was widely recognized (Bernardo, 1999; Meuwissen et al., 2001). Meuwissen et al. (2001) established the foundational principles of genomic selection. Their simulations used approximately 50,000 marker haplotypes derived from a limited number of phenotypic records within a simulated genome of 1000 cM, with markers spaced at 1 cM intervals. They found that predicting genetic values from markers could significantly accelerate genetic gain, especially when combined with reproductive techniques to shorten generation intervals. Their research also demonstrated that genomic selection could overcome the limitations of traditional MAS, such as its restricted variance capture, overestimated QTL effects, and discrepancies between mapping and breeding populations.

### 7.1 Evaluating genomic selection versus phenotypic and marker-assisted selection

The synthesis of findings across various studies highlights the superior performance of genomic selection compared to traditional phenotypic selection and MAS. Although genomic selection accelerates the depletion of genetic diversity, it consistently yields higher genetic gains per unit of time due to reduced breeding cycle durations and improved accuracy in parent selection. This advantage is particularly notable under conditions of low trait heritability and complex genetic architectures. Regular updates to training sets are essential to maintain the effectiveness of genomic selection, regardless of genetic architecture (Muleta et al., 2019; Lin et al., 2023). The integration of doubled haploid populations and high-throughput phenotyping further enhances the efficiency and stability of genomic selection, establishing it as a crucial tool in modern breeding programs. Despite its benefits, genomic selection accelerates the depletion of genetic variance compared to phenotypic selection.

Bernardo (1999) bridges the gap between MAS and genomic selection. He assessed the predictive accuracy of breeding values derived from phenotype data alone versus a combination of phenotype and markers. His findings showed that both methods were similarly effective in predicting untested single crosses, across a range of 10 to 100 QTL and trait heritabilities of 0.4 or 0.6. Bernardo and Yu (2007) further demonstrated that genomic selection provided a 6 to 43% greater response compared to marker-assisted recurrent selection, with this effect being more pronounced with a higher number of QTL and lower heritability.

Simulation studies consistently show that genomic selection outperforms phenotypic selection for traits with low heritability and polygenic architecture, such as yield, regardless of population sizes or selection cycles (Gaynor et al., 2017; Muleta et al., 2019; Ali et al., 2020; Silva et al., 2021; Lubanga et al., 2022; Chiaravallotti et al., 2023; Fritsche-Neto et al., 2024). This advantage is particularly evident in early selection cycles, with genomic selection reaching a performance plateau after fewer cycles (Gaynor et al., 2017; Muleta et al., 2019; Ali et al., 2020; Tessema et al., 2020; Lubanga et al., 2022; Chiaravallotti et al., 2023; Fritsche-Neto et al., 2024).

As expected, genomic selection leads to a more significant reduction in genetic diversity compared to phenotypic selection (Gaynor et al., 2017; Muleta et al., 2019; Tessema et al., 2020; Silva et al., 2021; Sabadin et al., 2022). This reduction is influenced by factors such as population size and genetic architecture but can be mitigated by retaining a larger number of individuals for future generations and incorporating new breeding materials from outside the program. Lin et al. (2023) found that parental populations of 15 exhibited the highest allele fixation, particularly when combined with single-seed descent and speed breeding. Larger population sizes can enhance selection intensity while preserving genetic variance, leading to significant genetic gains and reduced depletion of genetic variance (Muleta et al., 2019; Silva et al., 2021; Fritsche-Neto et al., 2024).

In practical breeding applications, the size of breeding populations is often constrained by limitations in phenotyping and genotyping capabilities. Silva et al. (2021) observed that the number of families and their sizes significantly impact genetic gains over 50 and 100 breeding cycles. More families contribute to increased long-term genetic gains, while larger population sizes are associated with enhanced short-term gains. Despite these constraints, genomic selection generally offers more advantages. Even when genomic and phenotypic selection yields similar genetic gains, the ability of genomic selection to bypass phenotyping costs often makes it preferable (Lin et al., 2023).

However, some simulation studies suggest that phenotypic selection can outperform marker-based selection strategies, particularly under epistatic genetic models and reduced genotype-by-environment interaction (Ali et al., 2020; Peixoto et al., 2023). Muleta et al. (2019) found that in oligogenic architectures—where a few genes with large effects control the trait—the number of genomic selection cycles affected short-term genetic gains. Their results indicated that while more cycles of genomic selection were beneficial for maximizing genetic gain, phenotypic selection achieved higher long-term gains before the fifth breeding cycle. Phenotypic selection also maintained greater genetic variance during early selection years, while prediction accuracy for traits with oligogenic architecture declined more rapidly than for traits with polygenic architecture (Muleta et al., 2019).

Addressing the enhancement of multiple traits simultaneously in plant breeding requires a comprehensive approach. The genomic selection index—a linear combination of genomic estimated breeding values leveraging genomic markers to predict net genetic merit and select parents from a non-phenotyped population—has proven effective in delivering greater genetic gains per unit of time compared to approaches based solely on phenotypic data (Ceron-Rojas et al., 2015).

Gaynor et al. (2017) utilized simulations to propose a two-part genomic selection program comprising: (i) a recycling component for rapid population improvement, and (ii) a product development component for generating commercial products and updating genomic models. Their study showed that this scheme more than doubled genetic gain compared to conventional breeding programs due to shorter breeding cycles. Similarly, Sabadin et al. (2022) and Fritsche-Neto et al. (2024) reported that genomic selection achieved genetic gains 0.5 to 6 times higher than phenotypic selection, attributed to reduced breeding cycle duration and enhanced selection accuracy. Tessema et al. (2020) and Li et al. (2022) reached similar conclusions, emphasizing that shorter breeding cycles were key to the improved effectiveness of genomic selection.

Breeding strategies utilizing genomic selection also demonstrated more stable genetic gains despite genotype-by-environment interactions, whereas traditional programs showed greater variability in genetic gains (Gaynor et al., 2017; Peixoto et al., 2023). This stability arises from using genomic predictions that incorporate multi-year historical data and markers designed to minimize genotype-by-environment interaction effects, ensuring optimal cohort selection (Bernal-Vasquez et al., 2022). Conventional programs, in contrast, rely on phenotypic data from a limited number of years. Bernal-Vasquez et al. (2022) showed that employing a kinship matrix to model breeding values over multiple years, even with single-year data, improved the outcomes in breeding programs.

Gaynor et al. (2017) also highlighted that applying genomic selection at the head-row or progeny stage enhances parent selection accuracy, crucial for successful breeding. Sabadin et al. (2022) demonstrated that applying genomic selection to F2 and evaluating F4 families phenotypically resulted in higher genetic gains compared to other methods, including exclusive phenotypic selection and rapid cycling from parental selection in F2. They also found that preserving a larger number of individuals (48-parent scenario) helped reduce genetic variance depletion and allowed for long-term genetic gains. Lin et al. (2023) supported these findings, noting that postponing line derivation to later generations is advantageous for traits with low heritability, offering a product development perspective. Silva et al. (2021) suggested that parental selection at the F4 stage is often treated as an early recycling generation due to limited and less reliable phenotypic data.

In summary, these authors advocate for the strategic use of genomic selection, emphasizing later-stage parental selection, for example, F4 generation (Gaynor et al., 2017; Silva et al., 2021; Sabadin et al., 2022; Lin et al., 2023). Their research highlights the benefits of this approach in terms of precision and long-term genetic gains while preserving a larger genetic pool. They argue that later-stage selection provides more stable gains and maintains genetic diversity better than early-stage selection.

In contrast, Li et al. (2022) found that selecting parents early in the breeding cycle, at stages such as F1 or F2, yielded substantially higher genetic gains compared to selection at later stages. They emphasize that earlier-stage genomic selection can yield higher immediate genetic gains, despite the moderate prediction accuracy. This result aligns with Peixoto et al. (2023) corroborated this by showing that early parental recycling, whether based on genomic or phenotypic selection, produced greater and faster genetic gains compared to scenarios where parental selection occurred after test cross-evaluation.

In summary, the synthesis of simulation studies emphasizes the advantage of selecting parents for recycling as early as possible while considering selective accuracy and trait-related breeding value estimates. Breeding programs equipped with high-throughput phenotyping, extensive genotyping, or other advanced methods can benefit from rapid-cycling strategies to maximize genetic gain. However, it is crucial to set specific accuracy or heritability targets for selecting parents to effectively address the complexities of breeding, such as specific objectives, available genetic diversity, and the balance between short-term and long-term gains.

For crops utilizing doubled haploids, rapid cycling within the population improvement component of two-stage breeding programs is particularly advantageous for managing heterozygosity and advancing generations efficiently. In crops where doubled haploid techniques are unavailable, it may be necessary to incorporate an additional stage with phenotypic assessments before yield testing to eliminate poorly segregating individuals and expedite the advancement of promising ones.

### 7.2 Evaluating different models in breeding value estimation

Best Linear Unbiased Prediction (BLUP) is fundamental in genomic selection, each offering unique advantages in optimizing prediction accuracy across complex genetic architectures. BLUP, utilized within mixed linear models, seeks to minimize bias by accounting for both fixed and random effects. On the other hand, Bayesian methods integrate prior knowledge with observed data, allowing for continual refinement of probability estimates to address uncertainty.

Meuwissen et al. (2001) provided early insights into these methods, demonstrating that while BLUP achieved a prediction accuracy of 0.73, Bayesian approaches, particularly those incorporating marker haplotypes, improved accuracy to 0.85. This early evidence underscored Bayesian methods’ potential to enhance genomic selection performance.

Subsequent research has shown comparable performance between BLUP-based methods (e.g., gBLUP, RR-BLUP) and Bayesian methods (e.g., BayesB, LASSO), with minor differences in accuracy and genetic gains across various models (Technow et al., 2012; Wang et al., 2015; Yao et al., 2018; Silva et al., 2021; Fernández-González et al., 2023). Bayesian approaches, particularly BayesB and Bayesian LASSO, have demonstrated superior accuracy and genetic gains in models accounting for dominance effects, especially when traits are influenced by fewer genes (Technow et al., 2012; Wang et al., 2018). BayesB, in particular, is noted for its effectiveness in capturing genetic gains and accelerating outcomes in early breeding cycles (Ramasubramanian and Beavis, 2021).

Certain BLUP variants, such as sBLUP (Super BLUP) and cBLUP (Compressed BLUP), demonstrate distinct advantages depending on the heritability of the traits. When compared to Bayesian LASSO and gBLUP in prediction accuracy, sBLUP outperformed these methods for Mendelian (simple) traits, whereas cBLUP proved more effective for traits with low heritability. In the cBLUP method, particularly for traits with low heritability, grouping individuals based on kinship and maximum likelihood (clustering) proved advantageous for estimating breeding values. This approach outperformed other well-known methods, likely due to replacing individual kinship with group kinship, which enhances estimation accuracy and mitigates the challenges associated with low heritability. However, it does not offer similar benefits under conditions of high heritability. In another study, RR-BLUP (Ridge Regression BLUP) demonstrated a slight advantage over other methods for traits influenced by numerous minor genes, making it a viable alternative (Wang et al., 2015).

Best Linear Unbiased Prediction (BLUP) is extensively applied in the estimation of breeding values, renowned for its simplicity and robustness in managing large populations with polygenic traits—characterized by the cumulative influence of numerous genes with small effects. A notable strength of BLUP is its capacity to minimize bias by simultaneously accounting for fixed and random effects, thereby delivering consistent predictions across varying scenarios. Specific adaptations, such as cBLUP, exhibit heightened efficiency when applied to traits with high heritability. However, BLUP is not without limitations. The method encounters challenges in accommodating complex genetic architectures, particularly those involving significant dominance effects, and exhibits reduced accuracy for traits with low heritability. In such cases, alternative approaches, such as Bayesian methods, often outperform BLUP. Furthermore, its reliance on additive genetic models restricts its adaptability to scenarios involving non-additive genetic interactions or traits controlled by a small number of major genes.

Bayesian methods offer an effective alternative for breeding value estimation, particularly for traits influenced by a few major genes or dominance effects, where they often outperform traditional approaches. Their flexibility allows the integration of prior knowledge and adaptation to diverse genetic models. Methods like BayesB and Bayesian LASSO are especially useful in early breeding cycles, capturing genetic gains efficiently. However, Bayesian methods are computationally intensive, which can be a limitation in large-scale breeding programs, and reliance on prior specifications may introduce bias if not properly defined. While Bayesian methods excel in specific genetic contexts, BLUP remains a reliable tool for polygenic traits with numerous small-effect genes, ensuring consistent performance in various breeding scenarios.

In conclusion, the choice between BLUP and Bayesian methods should be driven by the genetic architecture, population size, and specific breeding objectives. Bayesian methods, such as BayesB, are particularly beneficial for traits with fewer genes and in early breeding cycles. In contrast, BLUP methods are robust for traits with extensive QTL. RR-BLUP stands out for its flexibility and consistent performance across diverse conditions. Thus, no single model excels universally, emphasizing the need for tailored approaches based on the breeding program’s specific characteristics and goals.

### 7.3 Analyzing the impact of genetic parameters and architectures on gains

Increasing the size of breeding populations typically leads to greater genetic gains with genomic selection, provided that breeding objectives are well-defined and there is adequate. Extensive research has consistently indicated this trend (Lorenz, 2013; Muleta et al., 2019; Silva et al., 2021). However, it is important to note that the accuracy of genomic recurrent selection predictions tends to decline over generations. This decline is influenced by the trait’s genetic architecture and population size (Muleta et al., 2019). The population size can fluctuate due to various breeding operations such as truncation selection, which reduces genetic diversity and narrows the gene pool over generations. Additionally, other breeding operations, like crossing and inbreeding, may further impact the effective population size and the genetic variability available for selection. As these factors alter the genetic structure of the population, they can lead to reduced accuracy in genomic predictions, especially when selecting for complex traits with low heritability.

For traits with genetic architectures involving 40 or 400 QTL, the selection response often plateaus after approximately 10 to 15 cycles (Ramasubramanian and Beavis, 2021). Conversely, traits with more complex genetic architectures, such as those involving 4,289 QTL, can sustain selection responses for up to 30 to 40 cycles before encountering limitations (Ramasubramanian and Beavis, 2021). In this context, the numerous QTLs frequently exhibit significant interactions with the environment, ultimately reducing the heritability of the trait.

Heritability plays a critical role in determining genetic gain, with higher heritability leading to greater gains across various models and parental selection methods (Yao et al., 2018). Traits with high heritability demonstrate greater prediction accuracy, as a larger proportion of the phenotypic variation is attributed to genetic factors, and the corresponding QTL exhibit reduced interaction with environmental influences. In such cases, multi-trait analysis can improve the accuracy of breeding value predictions for traits with low heritability, particularly when these traits are correlated with high-heritability traits, as compared to single-trait analysis (Hayashi and Iwata, 2013). However, simulation studies investigating the advantages of multi-trait analysis remain limited, partly constrained by programming capabilities. To improve the accuracy of genomic selection, increasing the number of initial parents in the breeding cycle can be advantageous (Chiaravallotti et al., 2023). Modeling marker effects as population-specific is beneficial under low linkage disequilibrium and enhances prediction accuracy at lower marker densities (Technow et al., 2012). Additionally, incorporating dominance effects into models can substantially improve prediction accuracy, particularly for populations with convergent parentage (Technow et al., 2012). The effectiveness of genomic selection in introgressing large QTL is influenced by the trait’s genetic architecture, breeding strategy, and the number of initial parents, highlighting the complexity involved in optimizing genomic selection methods (Chiaravallotti et al., 2023).

### 7.4 Analyzing the impact of different training set configurations

The studies highlight the importance of optimizing training populations and marker densities in genomic selection to enhance prediction accuracy and genetic gain. In general, using lines in the test set that are closely related to the training population improves genomic breeding value (GEBV) accuracy by having a close relationship (Hickey et al., 2014; Muleta et al., 2019; Ramasubramanian and Beavis, 2021; Sabadin et al., 2022; Chiaravallotti et al., 2023). When closely related to the test set, smaller, well-structured training sets with low to mid-density markers effectively capture genetic information, especially with high linkage disequilibrium (Gorjanc et al., 2017). Regular updates to the training population with top-performing lines further boost genetic gain and prediction accuracy.

Expanding training and prediction sets with low-density genotyping and imputation is a cost-effective strategy to improve selection responses. Although low-density genotyping may initially reduce prediction accuracy, strategic imputation and training set updates can counter this, achieving results comparable to high-density genotyping. Targeting relevant populations enhances genetic relationships between training and prediction sets, leading to better prediction accuracy and breeding outcomes.

Simulation studies indicate that for biparental populations closely related to selection candidates, using 200–500 markers and about 1,000 phenotypes can achieve effective breeding value accuracy (Hickey et al., 2014; Gorjanc et al., 2017). Increasing marker density beyond 10,000 markers provides no additional benefit (Hickey et al., 2014). Phenotyping only a subset of doubled haploid (DH) lines is usually sufficient to predict the performance of the remaining lines. However, while studying single biparental population of doubled haploid lines, Lorenz (2013) found that including all DH lines in the training set significantly improves prediction accuracy compared to excluding part of them, suggesting that phenotyping a subset might be less effective. Budget constraints should guide these decisions.

Training sets with full-sibling and half-sibling families generally achieve high prediction accuracy (Melchinger et al., 2023a; Melchinger and Frisch, 2023b). Including unrelated families is discouraged due to potential negative impacts on prediction outcomes (Melchinger et al., 2023a; Melchinger and Frisch 2023b). To avoid these issues, unrelated families should be excluded, and a moderate number of genotypes per family (about 50) should be maintained. Training with full-sibling families, compared to half-sibling families, leads to greater selection gains in hybrid breeding programs, particularly where specific combining ability (SCA) effects are crucial.

There are two main strategies for genotyping: high-density genotyping, which is accurate but costly, and lower-density genotyping combined with imputation, which is more cost-effective yet retains acceptable precision. Gorjanc et al. (2017) found that accurate imputation is a cost-effective approach by (i) reducing genotyping costs per individual for both training and prediction sets and (ii) improving prediction accuracy by enlarging training sets. Using nearly 100 DHs, they reported imputation accuracies of 0.71 across families and 0.42 within families with high-density genotypes. Imputation led to a modest decline across families (from 0.71 to 0.66) compared to within families, from 0.42 to 0.34 (Gorjanc et al., 2017), likely due to the substantial genetic diversity represented and its connection to the training set. Accuracy improved most when imputed genotypes were used for both training and prediction sets (Gorjanc et al., 2017). For cross-family predictions, accuracy plateaued with marker densities between 100 and 50 low-density markers, while for within-family predictions, the plateau occurred between 200 and 100 low-density markers (Gorjanc et al., 2017).

Both modelling for cross-pollinated genomes, Gorjanc et al. (2017) and Do Vale et al. (2022) offer complementary perspectives on the trade-offs between low-density genotyping, imputation, and marker density in breeding programs. Gorjanc et al. demonstrated the effectiveness of expanding training and prediction sets through low-density genotyping and imputation, showing that the use of low-density genotypes, followed by imputation with as few as five segregating markers per chromosome, could achieve prediction accuracy and selection response comparable to high-density genotyping. They found that the response plateaued when 100 low-density markers were used, indicating diminishing returns with increased marker numbers. Imputation with about 20 segregating markers per chromosome still yielded satisfactory results in selection response and prediction accuracy, especially in non-phenotyped families, where genotyping with 50 low-density markers achieved a minimum accuracy of 0.3. In contrast, Do Vale et al. (2022) highlighted that increasing marker numbers can mitigate the decay of linkage disequilibrium (LD) over cycles, potentially improving long-term selection responses. They argued that while low-density marker panels offer benefits such as reduced multicollinearity, faster computation times, and cost-efficiency, high-density panels ultimately provide superior long-term gains, especially when the training set is not frequently updated. Their analysis suggests that the advantage of marker reduction through LD-based strategies is limited to a single cycle, as the accuracy of selection declines significantly in subsequent cycles, emphasizing the long-term value of high-density genotyping. Ultimately, the choice of marker strategy depends on the specific breeding objectives, resource constraints, and the need for sustainable improvements across multiple cycles.

Prediction accuracy is influenced by population structure, trait heritability, training set size, and the precision of genomic relationships at QTL regions. Regular updates are crucial for maintaining effective breeding programs (Hickey et al., 2014; Cuyabano et al., 2024). Research indicates that prediction accuracy typically declines within the first two cycles (Fritsche-Neto et al., 2024) and updating training sets generally enhances accuracy by preserving linkage disequilibrium between markers and QTL (Muleta et al., 2019; Ramasubramanian and Beavis, 2021; Sabadin et al., 2022; Chiaravallotti et al., 2023).

Trait heritability is a significant factor in the loss of prediction accuracy (Muleta et al., 2019; Ramasubramanian and Beavis, 2021). Ramasubramanian and Beavis (2021) found that genomic prediction models performed best when updated with data from up to 14 selection cycles, with optimal cycles varying by QTL count: 10 cycles for 40 QTLs and 30 cycles for 4289 QTLs. Results also depended on QTL effect sizes and whether effects were additive or dominant. Larger training sets, incorporating diverse conditions, offered more effective guidance for future selection decisions. Their study also revealed that static training sets when using genomic selection combined with a chain-rule mating design, can potentially capture up to 62% of the greatest genetic potential, even with advanced methods like RR-REML and Bayesian approaches (Ramasubramanian and Beavis, 2021). It worth mentioning that they considered 20 family designed with a common parent in all crosses with 100 F5 lines of family size. Expanding both the training population size and the number of replications improved prediction accuracy, with population size being particularly beneficial when genotyping costs were low, or heritability was high. Consistent with Lorenz (2013), phenotyping all doubled haploid (DH) lines resulted in greater genetic gains, making this approach preferable when feasible. Additionally, dispersing populations across multiple environments improved selection outcomes (Lorenz, 2013).

Sabadin et al. (2022) found that integrating top-performing parental lines from each cycle into long-term breeding schemes—while maintaining genetic diversity to balance allele frequencies and better fit the prediction model—yielded the highest genetic gains. They recommended retaining data from the most recent three generations to optimize genetic relationships between the training set and the target population. This method, referred as TSGPO, was noted for its ease of implementation and consistent performance.

Fernández-González et al. (2023) demonstrated that integrating test set information into the training set—referred to as targeted optimization—achieved the highest accuracies, particularly for low heritability traits like yield. For untargeted optimization approach, they advised that minimizing the average genetic relationship within the training set as a more reliable strategy than maximizing the relationship between the training and test sets. This finding is particularly noteworthy, as it challenges the widely accepted assumption that maximizing the relationship between training and test sets is essential for enhancing prediction accuracy (Wallace et al., 2018). Instead, it highlights that a universal approach may not be suitable in all cases.

### 7.5 Evaluation of different genomic selection strategies

Evaluating genomic selection strategies reveals a complex balance between short-term gains and long-term genetic potential. Rapid-cycling genomic selection programs, which initiate selection and parental recycling earlier, often surpass traditional phenotypic selection methods. This advantage arises from the reduced time required for parental recycling and the development of new cultivars, with both the rapid-cycling process and the genomic selection components contributing significantly to this efficiency. Advanced strategies such as Optimal Population Value Selection (OPV), Genomic Optimal Contribution Selection (GOCS), Optimal Haploid Value (OHV), and Weighted Genomic Selection (WGS) effectively preserve rare favorable alleles, achieve significant genetic gains, and ensure long-term genetic diversity, highlighting the trade-offs in optimizing breeding success.

Gaynor et al. (2017) proposed a two-part genomic selection program consisting of (i) a rapid population improvement component through genetic gain recycling, and (ii) a product development component that creates commercial products and updates genomic selection models with new data. Their research demonstrated that genetic gains were 1.31 to 1.46 times greater than those achieved with traditional genomic selection methods. The approach involving recycling parental lines from head-rows for population improvement yielded superior results, although these gains were inversely related to the depletion of genetic variability in subsequent seasons. Addressing this challenge will be critical for future strategies.

Gorjanc et al. (2017) tackled the challenge of balancing short-term and long-term genetic gains by proposing optimal cross-selection within rapid recurrent genomic selection programs. Their approach enhanced the efficiency of converting genetic diversity into genetic gain, reducing the loss of genetic diversity and the decline in genomic prediction accuracy that often accompanies rapid cycling.

Goiffon et al. (2017) found that the Optimal Population Value (OPV) method achieved superior genetic gains over the first ten generations compared to other methods. OPV and Genotype Building (GB) showed better long-term responses than basic genomic selection, WGS, and OHV. However, genomic selection and WGS provided rapid and significant improvements in GEBVs before reaching a plateau by generation 4, proving valuable in specific contexts.

OPV, GB, and OHV were more effective at preserving genetic variance within breeding populations over time compared to genomic selection or WGS. OPV, which evaluates the genetic merit of selection candidates rather than using truncation selection, is particularly useful for large, commercially significant cohorts. OPV and OHV also excel at maintaining rare favorable alleles, which might otherwise be underrepresented in training sets due to their infrequent occurrence or unique characteristics.

Beukelaer et al. (2017) compared Genomic Optimal Contribution Selection (GOCS), which limits genomic relationships among selection candidates, and Weighted Genomic Selection (WGS) with basic genomic selection. They found that GOCS and WGS achieved similar long-term genetic gains and inbreeding rates, but WGS slightly reduced short-term genetic gain compared to basic genomic selection. Alternative strategies such as IND-HE (focusing on heterozygosity) and IND-RA (focusing on rare alleles) demonstrated superior long-term gains and a better balance between genetic merit and diversity than GOCS or WGS, remaining effective across varying trait heritabilities and initial training population sizes. As outlined by Beukelaer et al. (2017), IND-HE aims to balance genetic gain and expected heterozygosity, seeking to control the inbreeding rate, defined as the relative decline in expected heterozygosity calculated from SNP markers. Additionally, IND-RA focuses on preserving rare alleles by incorporating them into the selection process.

Ramasubramanian and Beavis (2021) found that WGS produced greater responses in longer selection cycles compared to unweighted basic genomic selection, although it initially yielded lower response rates than phenotypic selection and basic genomic selection. They concluded that the effectiveness of genomic selection depends on the number of simulated QTL and trait heritability, with RR-BLUP offering better responses early on and WGS excelling in later cycles. Combining strategies can leverage different approaches’ strengths, mitigating costs, errors, and time requirements associated with phenotypic selection.

Akdemir et al. (2019); Moeinizade et al. (2019); Silva et al. (2021); Ramasubramanian and Beavis (2021), and Sabadin et al. (2022) contribute to the growing understanding of optimized selection strategies in breeding programs, offering insights into the balance between short-term genetic gains, long-term sustainability, and genomic advancements. Akdemir et al. demonstrated that multi-objective optimized parental proportion approaches, which balanced genetic variance and genomic estimated breeding values, yielded 20-30% higher outcomes over extended breeding cycles, outperforming index-based selection methods.

Silva et al. (2021) and Ramasubramanian and Beavis (2021) focused on selection methods within breeding cycles, highlighting the nuances of family-based selection. Silva et al. found that while across-family selection benefited short-term gains, within-family selection proved more effective for long-term improvements, with optimal results emerging at selection intensities between 7.5% and 10%. Their work emphasized that using 100 families with 150 individuals per family maximized genetic gains over ten cycles. Ramasubramanian and Beavis, in a similar vein, noted that genomic selection through RR-BLUP combined with recombination of high-merit parents within a hub network design was particularly effective in the first 5 to 10 cycles, further supporting the utility of family-based and genomic strategies for early-cycle improvements.

Building on these findings, Sabadin et al. (2022) refined genomic selection methods, demonstrating that a two-stage approach combining genomic selection in the F2 generation with phenotypic selection in the F4 produced 20% higher genetic gains than fast-recycling methods that skipped phenotyping. Their research highlighted the trade-offs between increasing selection cycles and the necessity of updating training sets to maintain selection effectiveness. This two-stage approach proved more advantageous than rapid cycling methods that relied solely on genomic selection in the early stages, reinforcing the importance of a balanced approach to maximize genetic gain over time.

In hybrid crop breeding, programs using high-density markers (10,000 or more) and QTL-level genotyping achieved greater genetic gains and heterosis compared to those using low-density and SNP markers (De Jong et al., 2023). Genomic approaches for predicting single-crosses were found to be more effective and simplified compared to rapid-cycling recurrent genomic selection and cyclical tester updates (Fritsche-Neto et al., 2024), though combined applications of these methods hold potential.

## 8 Multi-trait selection

Hayashi and Iwata (2013) and Akdemir et al. (2019) explore multi-trait genomic selection, offering complementary insights into improving prediction accuracy and genetic gains while managing program constraints. Hayashi and Iwata demonstrated that multi-trait Bayesian analysis, especially for low-heritability traits correlated with high-heritability traits, enhances genomic breeding value predictions. Although multi-trait analysis did not consistently outperform single-trait analysis for uncorrelated traits, its effectiveness in leveraging trait correlations makes it valuable for breeding programs targeting interrelated traits.

Akdemir et al. (2019) advanced this by incorporating multi-objective optimization frameworks, such as the MOOB approach, to achieve sustainable gains across multiple traits. Their method showed 20–30% higher long-term gains compared to traditional approaches like tandem and index selection. While Hayashi and Iwata focused on trait correlations, Akdemir et al. highlighted the importance of maintaining genetic diversity and optimizing parental contributions to ensure breeding population viability. The MOOB framework balances genetic variance, accuracy, and gains, addressing practical challenges such as the difficulty of assigning economic weights in index selection.

Both studies emphasize strategies that accommodate the complexities of multi-trait breeding. Hayashi and Iwata’s findings align with Akdemir et al.’s conclusion that methods exploiting trait interdependence or optimizing multiple objectives outperform traditional single-trait approaches. Moreover, the MOOB framework’s focus on genetic diversity preservation complements Hayashi and Iwata’s emphasis on accurate prediction, offering a comprehensive approach for breeding programs targeting long-term sustainability and trait improvement. Together, these approaches demonstrate the potential of advanced genomic tools and optimization techniques to address the multi-dimensional challenges of modern breeding.

## 9 Inbreeding, heterosis and epistasis

Studies highlight the challenge of balancing genetic gain with inbreeding in genomic selection, emphasizing the need for refined approaches. Beukelaer et al. (2017) found that genomic optimal contribution selection (GOCS) and genomic selection (GS) offer comparable long-term gains but struggle with inbreeding control. GOCS fails to achieve an optimal trade-off, while Goiffon et al. (2017) showed that weighted genomic selection (WGS) better controls inbreeding by targeting rare favorable alleles, offering a better balance. However, methods like GOCS and IND-OC lead to higher long-term gains but incur short-term penalties on progress.

Sabadin et al. (2022) noted that GS accelerates inbreeding in self-pollinated crops, depleting genetic variance more quickly than phenotypic selection. These findings underscore the need for updated training sets and strategies to preserve diversity while maximizing gains. GOCS and WGS each offer benefits but need continuous refinement to optimize long-term genetic improvement.

Advancements in genomic prediction models for hybrid breeding highlight the impact of marker density. De Jong et al. (2023) reported that high-density SNP markers generated 1.72 times more heterosis than low-density markers and 2.06 times more than QTL genotypes. Moreover, initial germplasm grouping is unnecessary to achieve high heterosis (Cowling et al., 2020). Models incorporating dominance effects improved heterosis by 1.44 times, emphasizing the value of capturing dominance interactions. Combining high-density markers with dominance-inclusive models enhanced prediction accuracy and genetic progress. Additionally, optimizing tester selection from previous cycles advanced genetic composition, complementing heterotic patterns (Melchinger and Frisch, 2023b).

Epistatic genetic architectures also affect selection strategies. Ali et al. (2020) showed that, under additive models, GS outperformed phenotypic selection (PS), but PS was more effective under epistatic models. Wang et al. (2004) found that dominance and epistasis slow genetic progress in wheat breeding, as epistasis stabilizes variance but hinders allele fixation. This suggests breeders should balance genomic and phenotypic approaches, integrating genetic architecture insights to optimize long-term selection outcomes.

## 10 Cost analysis

Hospital and Charcosset (1997) emphasized the benefits of combining foreground and background selection in marker-assisted backcross programs for introgressing quantitative trait loci (QTL). Pyramidal designs, where QTL are monitored sequentially, optimize genomic similarity with the recipient parent while minimizing costs when paired with appropriate population sizes. Kuchel et al. (2005) demonstrated the economic advantage of integrating marker-assisted selection (MAS) at the BC1F1 and haploid stages in wheat breeding, showing a 40% cost reduction and greater genetic gain compared to phenotypic methods. These findings highlight the importance of aligning genotyping and phenotyping for maximum efficiency.

In genomic selection, Lorenz (2013) and Hickey et al. (2014) focused on maximizing genetic gain through effective resource allocation. Lorenz’s simulations showed that increasing population size and phenotyping all DH lines maximized genetic gain under constrained heritability and genotyping costs. Hickey et al. suggested that combining phenotypic data from related populations improves prediction accuracy while reducing time and cost penalties. Recent advancements in low-density genotyping and imputation, as demonstrated by Gorjanc et al. (2017), can reduce genotyping costs by up to 87% with minimal loss in prediction accuracy. Using as few as 50 segregating markers per genome can yield a high return on investment (5.67 times the baseline), making genomic selection more accessible for early-generation selection.

## 11 Genotype-by-environment interaction

Studies by Buntaran et al. (2022) and Li et al. (2022) emphasize the need for refined selection strategies to address genotype-by-environment (GxE) interactions in genomic selection. Buntaran et al. showed that adjusting selection to environmental factors like drought or excess rainfall is critical for maintaining genetic progress, especially when environmental fluctuations bias breeding value estimates. Li et al.’s simulations focused on complex traits, such as grain yield and disease resistance, where GxE plays a significant role.

The impact of model parameters, selection intensities, and breeding strategies on long-term outcomes is further explored by Silva et al. (2021) and Covarrubias-Pazaran et al. (2022). Silva et al. highlighted GxE variance as a source of non-heritable variation, stressing the need for precise GS model parameterization to prevent genetic erosion. Covarrubias-Pazaran et al. simulated genotype-by-year and genotype-by-location interactions, offering valuable insights for improving program efficiency. However, better representation of GxE, particularly interactions across years, is still needed in simulation studies.

GxE models significantly impact genetic progress by enabling the use of environment-specific genetic performance. In the short term, they enhance trait prediction and optimize selection strategies for specific environments. In the long term, they support developing resilient germplasm with stability across diverse conditions. Strategies to leverage GxE include using selection indices tailored to environments, prioritizing traits with moderate to high heritability, incorporating genomic predictions, and conducting multi-environment trials to align GxE insights with breeding goals.

## 12 Software tools

Simulations are crucial in breeding programs, with several R packages offering comprehensive solutions for various needs. AlphaSimR is highly cited for its flexibility in modeling complex genetic architectures, selection schemes, and long-term strategies. Adam is essential for studies involving structured populations, tracking genetic inheritance in crossing programs. Synbreed excels in genomic prediction and analysis using SNP data. MoBPS is a modular and versatile tool for accurately simulating hybrid and genomic breeding programs. QU-LINE, a pioneer in simulating quantitative traits, remains valuable for modeling additive and epistatic effects in classical genetics. PedigreeSim specializes in simulating crosses and genetic recombination in structured populations, often used for inheritance and lineage management studies.

## 13 Conclusion and prospect

In plant breeding and genetics, simulations serve as mathematical models that replicate real-world biological conditions to address specific challenges and phenomena. Their growing importance stems from the necessity to optimize resource use in relation to program scale and genetic gains. The complexities of multi-trait selection make simulations crucial and challenging, as breeding pipelines are dynamic, influenced by business decisions, advancements in genotyping technologies, and fluctuations in resource allocation from year to year.

Simulations offer significant advantages by allowing researchers to explore diverse scenarios, genetic models, and methodologies, helping them identify the most effective strategies for developing target cultivars. These models facilitate the evaluation of genetic gain under various conditions, simulating entire or partial breeding programs. This capability enables comparisons of different breeding strategies by integrating genetic data, crossing schemes, propagation methods, population sizes, selection intensities, and the number of generations involved.

As breeding programs evolve and adopt new methodologies, the demand for simulations to assess their benefits and explore alternatives remains critical. Future research in this area is likely to focus increasingly on genetic diversity, pre-breeding efforts, and advances in genomic selection strategies. Simulations will continue to play a pivotal role in shaping the future of plant breeding by providing insights that help refine breeding pipelines and maximize genetic gains.

Studies highlight the need to align breeding strategies with trait heritability, selection timing, and genetic diversity, while accounting for genotype-environment interactions to minimize bias in early selection. Genomic selection enhances genetic gains by accelerating breeding cycles and optimizing parent selection, particularly for traits with low heritability and complex genetic structures. Regular updates to training sets are essential, irrespective of the genetic architecture. Bayesian methods are effective in early breeding stages with fewer genes, while BLUP is more suitable for traits with multiple QTL, and RR-BLUP offers flexibility across varying conditions.

Larger populations yield greater gains when clear objectives and appropriate germplasm are present. Updating with top-performing parentals improves accuracy and gains. Low-density genotyping, combined with imputation, provides a cost-effective alternative to high-density genotyping with comparable results. Evaluating genomic selection requires balancing short-term gains with long-term potential. Rapid-cycling programs are particularly effective.

Future research in plant breeding and genetics using simulations is poised to explore several promising avenues to enhance models and their applications. One key focus area may involve refining simulations to better capture genotype-environment interactions, particularly in the context of climate challenges.

Another potential direction for research is the expansion of simulations to support multi-genomic selection frameworks. As omics technologies continue to advance, future studies may aim to integrate multiple layers of data, such as epigenetics and transcriptomics, into simulations. This approach would establish a connection with the previously mentioned genotype-environment enhancement opportunity, while also contributing to a more comprehensive understanding of the mechanisms through which genetic networks influence trait expression.

There is significant potential in combining simulation models with artificial intelligence and machine learning algorithms. These technologies could be harnessed to optimize breeding pipelines by automatically identifying patterns, predicting outcomes, and dynamically adjusting selection criteria in real time. This approach would allow breeding programs to become increasingly adaptive and data-informed, facilitating faster genetic gains and greater breeding efficiency, with the primary focus on delivering value to farmers and consumers.

Finally, another critical focus is expanding simulations to support multi-trait selection frameworks, while refining key breeding parameters such as parental number, population size, and selection timing in both phenotypic and genomic programs. It is worth noting that the exploration of various genomic selection strategies remains a relevant and continually evolving area of research, as this approach becomes increasingly routine in breeding programs.
